# Error and anomaly detection for intra-participant time-series data

**DOI:** 10.1080/23335432.2017.1348913

**Published:** 2017-07-10

**Authors:** David R. Mullineaux, Gareth Irwin

**Affiliations:** a School of Sport and Exercise Science, College of Social Sciences, University of Lincoln, Lincoln, UK; b Cardiff School of Sport, Cardiff Metropolitan University, Cardiff, UK

**Keywords:** Biomechanics, kinematics, kinetics, outlier, statistics, variability

## Abstract

Identification of errors or anomalous values, collectively considered outliers, assists in exploring data or through removing outliers improves statistical analysis. In biomechanics, outlier detection methods have explored the ‘shape’ of the entire cycles, although exploring fewer points using a ‘moving-window’ may be advantageous. Hence, the aim was to develop a moving-window method for detecting trials with outliers in intra-participant time-series data. Outliers were detected through two stages for the strides (mean 38 cycles) from treadmill running. Cycles were removed in stage 1 for one-dimensional (spatial) outliers at each time point using the median absolute deviation, and in stage 2 for two-dimensional (spatial–temporal) outliers using a moving window standard deviation. Significance levels of the t-statistic were used for scaling. Fewer cycles were removed with smaller scaling and smaller window size, requiring more stringent scaling at stage 1 (mean 3.5 cycles removed for 0.0001 scaling) than at stage 2 (mean 2.6 cycles removed for 0.01 scaling with a window size of 1). Settings in the supplied Matlab code should be customised to each data set, and outliers assessed to justify whether to retain or remove those cycles. The method is effective in identifying trials with outliers in intra-participant time series data.

## Introduction

In data, there are often errors or anomalies, which collectively can be known as outliers. With the ever-increasing capability of technology, many trials are collected, but some of these trials contain errors that may arise from participant (e.g. a stumble during treadmill running) or experimental sources (e.g. misidentified kinematic markers). In contrast, some outliers will be anomalies that are real values, which could be worth exploring their source or assessing whether these rare events are beneficial or detrimental to performance. It is appropriate to always check for outliers (Osborne and Overbay 2004), and a primary justification is that regardless of whether the outliers are errors or anomalies they can have a substantial effect on many statistical analyses of the data. For instance, the standard deviation (SD) has a breakdown point of 0, which is the proportion of errors whereby the estimator still provides a good indication of the original distribution (Hampel 1971), and hence, in the presence of a single outlier, the SD estimator can provide a non-robust measure of variability.

The number of outliers to expect in a data set is unknown, although often it is intended that there are none or only a few outliers. Quantifying a ‘few’ is not possible, as it would depend on the quality and quantity of data, and on the research question. The justification to remove outliers has many advocates (e.g. Osborne and Overbay 2004) and opponents (e.g. Orr et al. 1991), and hence, identification of outliers does not mean any should be deleted and the rationale to retain or remove outliers should be clearly described.

In essence, an outlier increases the variability in the data. Data transformations are often used in time-series analyses to reduce variability between trials or between participants, which in turn will typically lead to fewer outliers. A requirement for many time-series analyses is that the data are of the same length, hence data are often time normalised when multiple trials are collated to create the average trial or to conduct time-series analysis (e.g. vector coding, Tepavac and Field-Fote 2001; CI2, Mullineaux 2017; and; outlier detection, Sangeux and Polak 2014). On the assumption of a ‘typical’ time-series for each participant, this time-normalising should lead to a reduced temporal variability and, where the temporal variability is still high, rectification or time-warping has been proposed (Kale et al. 2003). Where there is spatial variability offset normalisations have been demonstrated (Mullineaux et al. 2004), many of which are based on parametric assumptions, and hence, it can be valuable to remove outliers prior to applying such modification and other analytical techniques.

There are many methods for identifying outliers, and in a review of these, it is concluded that the choice is dependent on which is most suitable for the problem (Hodge and Austin 2004). Biomechanical data are often analysed one-dimensionally (1D; i.e. the spatial aspect). For 1D data, as parametric based statistics (e.g. SD) are ineffective in detecting outliers, it has been proposed outliers are defined as ±2.5 × 1.48 MAD (median absolute deviation) away from the median (Leys et al. 2013; where 1.48 approximates MAD to the SD).

As it is common in biomechanics to collect time-series data, these data can be analysed in two dimensions (2D) to explore the spatial–temporal components (i.e. visually, the variable-time axes of a plot). Two types of 2D outlier detection methods have been ‘shape’ and ‘window’ based. Shape methods explore the entire cycle to assess the fit of the shape between all the cycles, which have been applied to biomechanical data (e.g. Arribas-Gil and Romo 2014; Sangeux and Polak 2014). Window methods explore outliers across fewer points in the time series, such as using a moving-window SD (mwSD; Brownlees and Gallo 2006), but these have not incorporated a criterion component and have not been applied to biomechanical data. In biomechanics, multiple trials from a single participant provides the basis to create a criterion (e.g. median or mean), which could be used in a window based method to assess deviations from in identifying outliers.

Identifying 1D and 2D outliers could be achieved in two stages. Stage 1 would identify 1D spatial outliers at each time-point (e.g. ±*α* 1.48 MAD, where *α* is the significance level-based scaling factor), and stage 2 would take account of the influence of a range of time points on each other to identify 2D spatial–temporal outliers (e.g. ±*α* mwSD). As the two stages, and particularly the criterion time series, can be susceptible to variability between subjects, such a method would only be suitable for intra-participant trials. Hence, the purpose of this study was to propose a two-stage method of detecting trials with single-point outliers in intra-participant time-series data.

## Methods

The test data were taken from a previous study (Mullineaux et al. 2013) where all procedures were approved by the institution’s ethics review committee. In summary, data were obtained for six healthy recreational runners (height 1.72 ± 0.09 m; mass 74 ± 15 kg) running at 3.35 m/s (7.5 mph) on an AlterG treadmill (P200; AlterG, Fremont, CA, USA) and on a dual-belt standard treadmill (TM-09-P; Bertec, Columbus, OH, USA). Four-marker rigid-shell clusters on the shanks and thighs (Cappozzo et al. 1997) and four markers on the foot (heel, lateral posterior, first and fifth metatarsal) were captured at 200 Hz via eight cameras (4 × Eagle and 4 × Eagle-4) and recorded using Cortex software (v2.0; Motion Analysis Corporation, Santa Rosa, CA, USA). Three 30 s conditions were recorded: 40% and 100% of body weight on the AlterG treadmill, and normal running on the standard treadmill.

Data were analysed in Matlab (v2015a; Mathworks, Natick, MA). Data were smoothed using a fourth order Butterworth filter with a 6 Hz cut-off frequency. Heel-strike was defined as the maximum forward excursion of the toe marker, and each stride from heel-strike to subsequent heel-strike of the same leg (0–100% of stride time) were extracted and time normalised to 101 time points using a cubic spline interpolation. The thigh, shank and foot three-dimensional joint coordinate systems (Grood and Suntay 1983) were calculated. From the joint coordinate systems, only angles in the sagittal plane were calculated, which were normalised to the anatomical standing position (i.e. 0° is angle during standing) for knee flexion–extension (negative flexion; positive extension) and ankle dorsi-plantar flexion (negative dorsiflexion; positive plantarflexion). In addition, the vertical displacement of the mid-knee virtual marker was calculated.

Incorporating both 1D and 2D aspects, a two-stage outlier detection method was applied to each participant’s data separately in stage 1 and stage 2 described below. This was calculated using Matlab code (Appendix 1) that outputs the data with the outliers removed, but it also provides lists of the trial numbers kept and removed so that these trials can be explored to determine if they were appropriately removed.

Stage 1 (Equation (1)):

• Calculate median absolute deviation confidence interval (MADCI). At each time-point *p*, MAD was calculated and scaled by multiplying by 1.48 (to approximate MAD to the SD) and by *t*_*α*1_ (to scale to varying confidence interval sizes using the *t*-statistic to account for the number of cycles);
• Remove outlier cycles,where a data value in cycle *j* at time point *p* exceeded the limits of the ‘median at *p*’ ± MADCI_*p*_ the entire cycle was removed.

(1) $${\mathrm{MADCI}}_{p}=t_{\propto 1}1.48\left(\mathrm{median}(\left|x_{p(j=1\mathrm{to}k)}-\mathrm{median}\left(x_{p}\right)\right|\right)$$

$$\{\mathrm{median}\left(x_{p}\right)-{\mathrm{MADCI}}_{p}>{\text{median}}_{pj}>\mathrm{median}\left(x_{p}\right)+{\mathrm{MADCI}}_{p};\;\mathrm{stride}\;\mathrm{outlier}$$

where: MADCI_*p*_ is median absolute deviation confidence interval at *p*; *p* is time point (i.e. 1 to *n*); *n* is total number of time points (e.g. *n* = 101); *t*_*α*1_ is the two-tailed *t*-statistic for given *α*1 and degrees of freedom (df) for the number of cycles (df = *k* − 1); *α*1 is significance level (which can be any value between 0 and 1, but 0.01, 0.001 or 0.0001 were selected as 0.01 is a minimum recommended by Leys et al. (2013), and 0.001 and 0.0001 are more stringent levels that would lead to fewer outliers being detected of benefit when fewer outliers are expected or desired); 1.48 is a constant to approximate the MAD to the SD; *j* is cycle; *k* is total number of cycles, and; *x* is variable.

Stage 2 (Equation (2), and expanded in Equation (3)), using a moving-window of size *b*:

• Pad data. At the start (and end) of each cycle, the data were padded by *b* time points using reflection of the first *b* (and last *b*) time points. This allows the moving window to be calculated for all n points. Padding is based on the data trend, and hence, this precedes detrending;
• Detrend data. For each cycle, the mean cycle was subtracted to reduce the spatial variability between strides;
• Calculate moving-window confidence interval (mwCI). For each time point, with a window of *b* time points either side, the mwCI_*p*_ was calculated across the cycles and scaled by multiplying by *t*_*α*2_ (to scale to varying confidence interval sizes using the *t*-statistic to account for the number of cycles);
• Remove outlier cycles. Where a data value in cycle *j* at time point *p* exceeded the limits of the ‘mean at *p*’ ± mwCI_*p*_, the entire cycle was removed.

(2) $${\mathrm{mwCI}}_{p}\;=\;t_{\propto 2}\mathrm{SD}\left(x_{mj}\right)\{{\overset{¯}{x}}_{p}-{\mathrm{mwCI}}_{p}>x_{pj}>{\overset{¯}{x}}_{p}+{\mathrm{mwCI}}_{p};\;\;\mathrm{stride}\;\mathrm{outlier}$$

(3) $${\mathrm{mwCI}}_{p}\;=\;t_{\propto 2}{\left(\frac{1}{k\left(2b+1\right)-1}\sum_{m=p-b}^{p+b}\sum_{j=1}^{k}{\left(x_{mj}-\frac{1}{k\left(2b+1\right)}\sum_{m=p-b}^{p+b}\sum_{j=1}^{k}x_{mj}\right)}^{2}\right)}^{0.5}$$

where in addition to notation for Equation (1): mwCI_*p*_ is moving-window confidence interval at p; *t*_*α*2_ is the *t*-statistic for given *α*2 (either 0.01, 0.001 or 0.0001); *m* is index of time point around *p*, and; *b* is moving-window size (either 0, 1, 2 or 3, equivalent to up to approximately ±3% for data time normalised to 101 data points). Where *b* = 0, the SD is calculated for the data at only time point *p*.

The two-stage outlier detection method was tested for all 36 combinations of *α*1 and *α*2 (0.01, 0.001 and 0.0001, approximating to *Z* scores of 2.6, 3.3 and 3.9) and *b* values (0, 1, 2 and 3). These were each applied to 108 sets of data, which were 6 participants, 2 legs, 3 conditions (40% BW and 100% BW on the AlterG treadmill, and 100% BW on the standard treadmill) and three variables (ankle angle, knee angle, vertical knee displacement).

**Figure 1.**
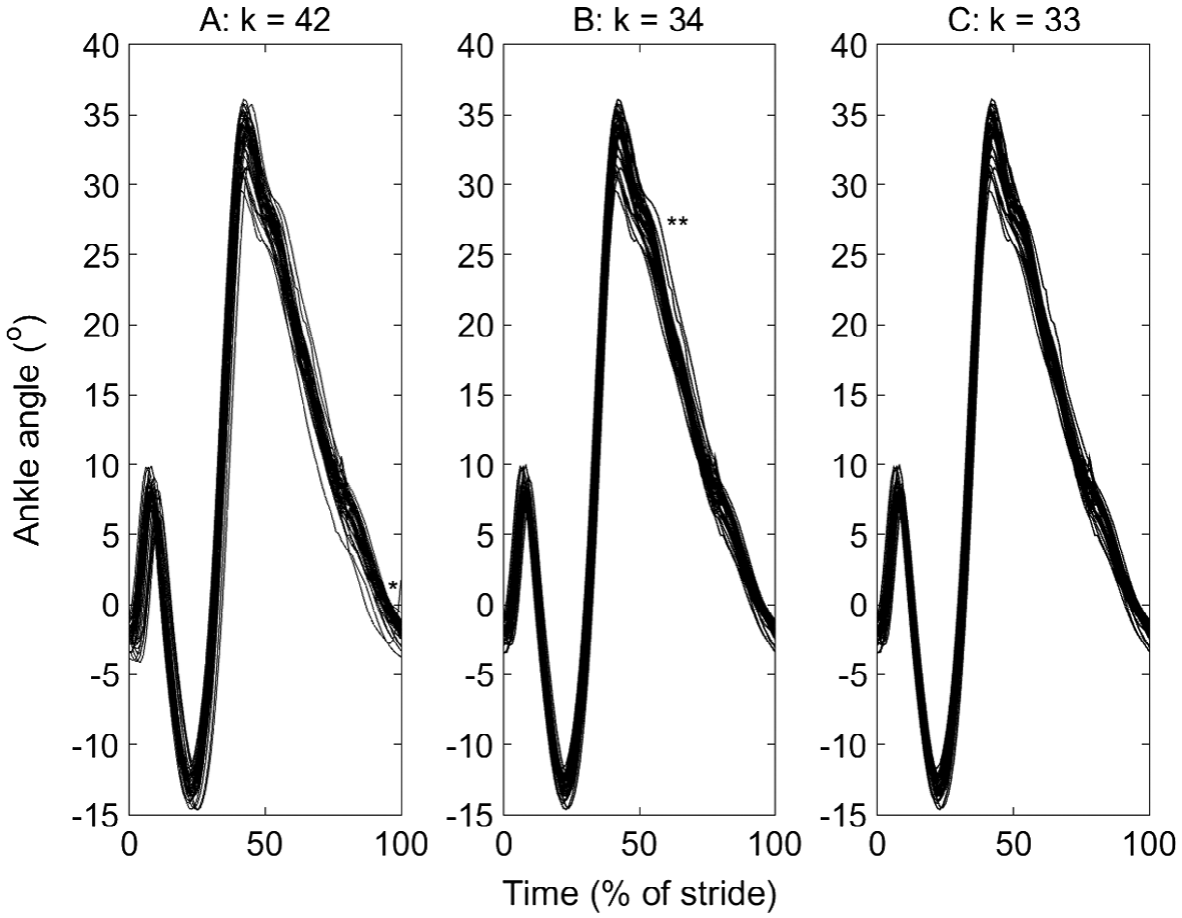
Ankle plantar-dorsi flexion angle for one participant on a standard treadmill running at 3.35 m/s: (a) before, (b), after stage 1 (b; *α*1 = 0.0001) and (c) after stage 2 (*α*2 = 0.01; *b* = 1) of a two-stage outlier removal method. The data were the right ankle from heel strike (0%) to heel strike (100%). The stance phase is approximately 0–40%. A potential spatial (*) and spatial–temporal (**) outliers are indicated, which are removed, and the number of cycles at each stage are indicated (*k*). The cycles that were deleted are listed here for stage 1 (3, 4, 5, 10, 26, 27, 41, 42) and stage 2 (11), which correspond to the column numbers in the supplementary material used to create this figure, to facilitate comparison with other outlier detection methods.

The data were presented for the angles and displacements separately. Calculations were performed on each time point (i.e. *p* = 1 to 101) and at each stage (raw, stage 1, stage 2) for all 36 combinations of settings for all 36 displacement trials and 72 angle trials. First, the normal distribution was tested using the Lilliefors test (*p* > 0.05), and the count of the number of time-points at each stage that were not-normally distributed was calculated (ideally count = 0). Second, the count of the number of cycles at each stage was calculated. Third, the descriptive statistics of the data at each stage were calculated (means and SD). These calculated variables at each setting at Raw to Stage 1, Raw to Stage 2 and Stage 1 to Stage 2 were compared using paired *t*-tests at a statistical significance level of 0.05. The data were presented as the means for the raw data, then the reductions in these to stage 1 and then the further reductions to stage 2. The total number of statistically significant settings from each stage to the next were counted. The raw data for one variable for a single subject, which was used to create Figure 1, are provided in the supplementary material to enable researchers to compare the results between this and other outlier removal methods.

## Results

An example data set that contains both one-dimensional and two-dimensional outliers are illustrated (Figure 1(a)). This trial contains a high number of outliers, and although there are no extreme values, some of the one-dimensional outliers maybe errors. For example, the 1D-outlier trial (indicated in Figure 1(a)) at the end of the stride that increases in contrast to all the other trials decreasing maybe an error from inaccurate toe-off detection. Following stage 1 with *α*1 = 0.0001, eight cycles were removed (Figure 1(b)). Following stage 2 with *α*2 = 0.01 and *b* = 1, one further cycle that was unrepresentative of the remaining general time series was removed (Figure 1(c)). The remaining cycles’ mean scores possess almost the same mean as the raw data, and although two of the 36 settings for the mean angle data did reduce significantly (*p* < 0.05), these two reductions were in the presence of multiple severe outliers. The maximum mean changes were 1.3° and  <0.001 m. The principal changes were in the normal distribution and variability of the data (Table 1 and Table 2). For the same *α*2, greater *b* typically resulted in a larger reduction in the count of outliers and SD of the data, but the values were small (maximum changes of 0.3 count; 0.03°; <0.001 m).

**Table 1.** Reduction in number of cycles, variability and normal distribution of lower limb angle data before (Raw) and after a two-stage time-series outlier removal method (stage 1 and stage 2).

Stage	Settings			Cycles (count)		SD (°)		Normal (count)	
	*α*1	*α*2	*b*	Mean		Mean		Mean	
Raw				38.2		11.9		22.6	
				Reduction	Sig	Reduction	Sig	Reduction	Sig
Stage 1	0.01			12.3	Y	10.3	N	16.0	Y
	0.001			6.0	Y	10.0	N	13.0	Y
	0.0001			3.5	Y	9.7	N	10.6	Y
	3 sets				3		0		3
Stage 1 to 2	0.01	0.01	0	0.5	Y	0.2	Y	0.4	N
	0.001	0.001	1	<0.1	N	<0.1	Y	0.1	N
	0.0001	0.01	1	2.6	Y	0.4	Y	1.3	Y
	0.0001	0.0001	3	0	N	<0.1	N	0	N
	36 sets				17		22		5
Raw to 2	36 sets				36		0		36

Notes: The data are for six participants × 3 conditions × 2 sides × 2 angles; stage 1 has three sets of statistical significance levels (*α*1 = 0.01, 0.001 and 0.0001), all presented; stage 2 has 36 sets of *α*1, *α*2 (0.01, 0.001 and 0.0001) and moving-window size (b = 0, 1, 2, and 3) but only four sets covering the least to most stringent combinations are presented; statistical significance (Sig) indicated as no (N; *p* > 0.05) or yes (Y; *p* < 0.05), and the numbers indicate the total of the three sets (stage 1) or 36 combinations of sets (stage 1 to stage 2, and raw to stage 2) that were statistically significant.

**Table 2.** Reduction in number of cycles, variability and normal distribution of knee vertical displacement data before (Raw) and after a two-stage time-series outlier removal method (stage 1 and stage 2).

Stage	Settings			Cycles (count)		SD (°)		Normal (count)	
	*α*1	*α*2	*b*	Mean		Mean		Mean	
Raw				38.4		0.01		19.1	
				Reduction	Sig	Reduction	Sig	Reduction	Sig
Stage 1	0.01			11.3	Y	0.003	Y	13.9	Y
	0.001			5.3	Y	0.002	Y	10.4	Y
	0.0001			3.0	Y	0.002	Y	8.1	Y
	3 sets				3		3		3
Stage 1 to 2	0.01	0.01	0	0.3	Y	<0.001	Y	0.1	N
	0.001	0.001	1	<0.1	N	<0.001	N	0	N
	0.0001	0.01	1	2.2	Y	<0.001	Y	1.3	Y
	0.0001	0.0001	3	0	N	0	N	0	N
	36 sets				12		12		6
Raw to 2	36 sets				36		36		36

Notes: These data are for six participants × 3 conditions × 2 sides; stage 1 has three sets of statistical significance levels (*α*1  =  0.01, 0.001 and 0.0001), all presented; stage 2 has 36 sets of *α*1, *α*2 (0.01, 0.001 and 0.0001) and moving-window size (b  =  0, 1, 2 and 3) but only four sets covering the least to most stringent combinations are presented; statistical significance (Sig) indicated as no (N; *p* > 0.05) or yes (Y; *p*  <  0.05), and the numbers indicate the total of the three sets (stage 1) or 36 combinations of sets (stage 1 to stage 2, and raw to stage 2) that were statistically significant.

In assessing the distribution of the data at each of the 101 time point, ideally there would be 0 not-normally distributed. With respect to the angles data, the mean number of data points that were not-normally distributed was 22.6 (raw) that reduced by between 8.1 and 16.0 (stage 1), and by a further 0–0.4 depending on the combination of setting used (stage 2).

The variability of the data reduced substantially in both the angles and displacement data. Firstly with the angles data, for the 36 combination of settings, between 3.5 and 12.3 cycles were removed from the mean of 38.2 cycles across the 72 angle trials. From the most stringent settings (i.e. *α*1 and *α*2 = 0.0001, and moving-window size of *b* = 3) to the most lenient (i.e. *α*1 and *α*2 = 0.01, and moving-window size of *b* = 0), the variability reduced similar amounts at stage 1 and reduced less at stage 2. Similar results were found for the 36 displacement trials.

In reviewing the angle data in more detail, there are clear outliers. With the most stringent setting of *α*1 = 0.0001 at stage 1, which is least likely to remove data, the raw mean of 38.2 cycles reduced by 3.5 cycles and the SD of 11.9° reduced by 9.7°. Regardless of such a large reduction in SD, this change was not statistically significant (*p* > 0.05). Owing to the normal distribution violation, the statistical significance testing of a paired *t*-test in this instance was also unable to detect such a large mean change of the SD being statistically significant for all three settings of *α*1 for raw to stage 1 comparisons. This problem also exists in comparing the raw to stage 2 data, where there were no overall statistical significant reductions in SD. In contrast, from stage 1 to stage 2, the SD reduced by only 0.4° or less, and yet 22 of the 36 combinations of settings with these small SD reductions were statistically significant (*p* < 0.05).

With the displacement data, raw to stage 1 for all three *α*1 settings resulted in SD reductions of between 0.002 and 0.003 m that were statistically significant (*p* < 0.05). This statistical significance for small changes emphasises the data had fewer extreme outliers. At stage 2, SD reductions were less than 0.001 m, yet for 12 of the 36 settings these small reductions were statistically significant (*p* < 0.05).

## Discussion

This study describes a method to detect spatial (stage 1) and spatial–temporal (stage 2) outliers that exist at single points along the time series of intra-participant data. For stage 1, in previous research, it is recommended to use a scale factor of 2.5 (Leys et al. 2013), which approximates to a *Z*-score for *α* = 0.01. In this study, rather than the *Z*-score the *t*-statistic is proposed that accounts for the number of cycles in the degrees of freedom (df), and the scale factor, for example, for *α* = 0.01 for df = 37 is *t*_*α*_ = 2.7. Still, using *t* for *α*1 = 0.01 at stage 1 results in a mean of 12.3 cycles being identified, which is problematic. As a minimum, it is suggested t for *α*1 = 0.0001 is used in stage 1 and that it is advisable to inspect the data by comparing pre- and post-plots to determine if appropriate cycles have been removed. The desired outcome would be to identify all error outliers that should be removed and assuming normally distributed data that none or a few anomaly outliers identified that should only be removed if appropriately justified. Separating these two forms of outliers is difficult, hence as a compromise the method should result in only a few trials being removed, which can be achieved by adjusting the *α*1, *α*2 and *b* settings based on the specific situation each time. As mentioned previously, a ‘few’ cannot be defined and researchers need to justify what is an appropriate number of outliers to remove with each data set.

There are cautions in using any outlier detection method. Nevertheless, the data do emphasise that outliers exist, which have a large effect on the data. The angle data clearly obtained outliers which were errors, which was observed by visual inspection of the plots and the numerical results. For instance, the SD of 11.9° reduced to 2.2° for *α*1 = 0.0001 following removal of a mean of 3.5 cycles at stage 1, yet this SD reduction was not statistically significant. In the presence of outliers, particularly for small samples sizes, the SD is misrepresentative of the spread of the data. Indeed, this emphasises the need for stage 1 to be based on a method that does not use parametric based assumptions, such as the SD, as it would not detect outliers. Hence, the stage-1 equation based on the non-parametric-based MAD was appropriate.

Following the removal of outliers at stage 1, less stringent settings at stage 2 can be used. Even with *α*2 = 0.01 and moving window of *b* = 1, fewer than a mean of 0.5 cycles were identified. Although fewer cycles were removed, it is proposed stage 2 is important in detecting two-dimensional outliers, such as small time shifts in the data, which may impact upon subsequent analyses and interpretation. Time shifts can be removed through other techniques such as rectification (Kale et al. 2003), but instead of transforming data where there are small temporal differences the outlier-detection method might provide a simpler solution to correcting temporal outliers. This solution to removing outliers may be valuable for both the researcher and practitioner as the data analysis would be simpler, and the average of the remaining cycles would provide a more valid representation of the ‘typical’ movement rather than being a distorted or ‘mythical average’ of the movement (Dufek et al. 1995).

The primary assumptions in the method proposed are that the cycles possess similar patterns (at stage 1 and stage 2) and a normal distribution (only at stage 2). Stage 1 is less susceptible to similarity of cycles, but in stage 2 where features such as subtle differences in local maxima, varying gradients, or cycle patterns that are offset from the group, the larger moving window will result in identification of slightly more outliers. Hence, the more varied the data the smaller the moving-window size that should be used in stage 2. Stage 1 is not underpinned by the normality distribution, although it leads to improving this distribution which is necessary as stage 2 uses the mean and SD that needs to more closely meet this assumption of normality. In general, the method is restricted to similar trials that would be likely from intra-participant data. Data with greater variability should be corrected for by using more stringent criteria (i.e. smaller *α*), which would lead to the identification of fewer outliers.

The pre-processing of the data can reduce the presence of outliers. The sample data used here were smoothed that may reduce spatial-outliers, and the time normalisation may reduce temporal outliers. In addition, the outlier calculation method includes an offset normalisation, which may reduce spatial outliers. These processes can be omitted if it is considered more beneficial to the research process. Further, although outliers may not exist in raw data (e.g. displacement), they may exist in calculated data from either multiplication of errors in combining raw data or through the specific method used (e.g. joint coordinate system used here to create joint angles), and hence, it is recommended outlier identification is performed on all created variables.

As stage 1 of the method is based on a non-parametric statistic, it is less susceptible to distribution assumptions, but as stage 2 is based on a parametric statistic, there is a greater need to consider the number of trials required. In a technique exploring the bivariate plots of intra-participant data (Mullineaux 2017), it is recommended that a minimum of 10 trials are used as this leads to a sufficiently low bias between the actual area and ellipse quantified area (Jackson et al. 2011). Given the complexity of assessing how many trials are required in biomechanical research, and independently of the number of subjects as applicable to this situation on intra-participant data, for typical simulation criteria a total of 9 ± 8 trials are required (mean ± 95% confidence intervals; Forrester 2015). Consequently, it is proposed there is no set minimum, although approximately nine trials might be advisable to use for this outlier detection method.

The primary benefit of applying an outlier detection method is to remove data containing errors (e.g. incorrectly identified markers). It may also remove anomaly or unusual trials that might represent natural variation. Removing both types of outliers will lead to improved distributions, such as the normal distribution necessary for subsequent analyses. In contrast, research where the variability is explored such as in studying reliability or functional-variability (e.g. Preatoni et al. 2013), then removing ‘unusual’ trials maybe detrimental to the principles of the research. Nevertheless, many of the analyses in reliability and functional-variability research are underpinned by the normal-distribution assumption, or the more complex analyses include first and second derivatives that would magnify the difference from the group. Hence, it is proposed that outliers should be removed to lead to more robust and valid analyses in applying more complex analyses. The outlier method resulted in only small and non-significant changes to the mean values, and both large and small significant changes to the SD values.

## Conclusions

This study describes an outlier detection method, which has been demonstrated to identify outliers in both raw (i.e. displacement) and calculated data (i.e. angles). Potentially outliers detected in the raw data will improve the calculated data, but as it is important to improve the normal distribution on the data that is analysed, it is recommended that outliers are also detected, and potentially removed, on the calculated data. The improvement in the normal distribution found is valuable, as this is an assumption underpinning the valid use of many analytical and statistical techniques. Even if there are known errors, and a few likely anomalies that it is desirable to remove for statistical reasons, it is cautioned that more stringent settings should be used (e.g. *α*1 ≤ 0.0001 at stage 1) particularly where research is concerned with reliability or functional–variability themes. Whichever settings are used, these should be individualised to the specific data set so that only a few outliers are detected, where the appropriate number is judged by the researcher, and only then delete outliers if they are apparent and justified. The Matlab code provided identifies the trials that are detected as outliers so that they can be explored. If, for instance, there are many outliers and the data are not normally distributed this may suggest keeping all the trials and changing the subsequent analysis to a non-parametric based approach. Further, alternative outlier removal methods may be more appropriate, and the raw data from Figure 1 is provided in the supplementary material to enable different outlier removal methods to be compared. In summary, the two-stage outlier detection method is effective in identifying trials with single-point outliers in intra-participant time-series data.

## Disclosure statement

No potential conflict of interest was reported by the authors.

## Supplemental data

Supplemental data for this article can be accessed at https://doi.10.1080/23335432.2017.1348913

## Supplementary Material

TBBE_1348913_Supplementary_material.xlsx

## Appendix 1

**Table:**

function [x2D,trialskept1,trialskept2]=ts2Doutlier(xraw,b,alpha1,alpha2)
% input: xraw data over time in rows (all equal length); k trials in columns
% input: b is moving-window size, e.g. 0 or 1 or 2 or 3
% input: alpha1,alpha2 p values for 1D,2D stages (e.g. 0.0001,0.01)
% output: x2D is x with 1D and 2D outliers removed
% output: trialskept1 & 2 is list of trial numbers kept after stages 1 & both 1and 2
% toolboxes required: image (padarray; std2); statistics (mad; tinv)
% Mullineaux DR, Irwin G. 2017. Error and anomaly detection for intra-participant time-series data. % Int Biomech. 4:28–35. doi:https://doi.org/10.1080/23335432.2017.1348913
[n,k]=size(xraw); %n number time points; k number of trials
trialskept2 = 1:k; %numbered list of k trials, to keep record of trials kept
x1D = xraw; %create x after 1D outliers removed (temporary as copy of xraw)
%==========================================================================
% 1D outlier: identify and remove columns/trials with any 1D outliers
t1 = abs(tinv(alpha1/2,k-1)); %2-tailed t-statistic for alpha1 and k-1 df
for p = 1:n % loop through each point p
xloop = x1D(p,:); % temporary x for the loop
medianpj = nanmedian(xloop); % median at point p for all j = 1:k trials
MADCI = t1*1.4826*mad(xloop,1); %MAD CI (1.4826 makes MAD~ = 1SD)
lower = medianpj-MADCI; % lower and upper limits
upper = medianpj+MADCI; % below find outliers outside limits …
x1D(p,xloop<=lower | xloop>=upper)=NaN; % … and replace with NaN
end
nanCt = sum(isnan(x1D),1); % sum NaNs in each column
trialskept2(:,nanCt>0)=[]; %reduce list of trials to trial numbers kept
trialskept1 = trialskept2; %reduce list of trials to trial numbers kept
x1D(:,nanCt>0)=[]; % removes columns with any NaNs (outliers) to leave x1D
%==========================================================================
% 2D outlier detection; start with padding data by *b* samples at each end
xpad=padarray(x1D,b,’symmetric’); %temporary x padded using reflection
[nb,k2]=size(xpad); %rows is pb = *n* + 2**b*; columns is k2<=k (trials for x1D)
% ———- % detrend data (by removing mean cycle)
xpadmean=mean(xpad,2); % create mean cycle
xpadmean=repmat(xpadmean,1,k2); % make matrix same size as xpad
xpaddetrended=xpad-xpadmean; % remove mean cycle from all cycles
% ———- % create moving SD for all (i.e. 1 column)
xpadmwSD=nan(nb,1); % create correct size matrix with NaNs
for m=b+1:n+b % loop to incorporate moving-window index m (j index in std2)
startm=m-b; % start row of index m
endm=m+b; % end row of index m
xpadmwSD(m,1)=std2(xpaddetrended(startm:endm,:)); % calculate moving SD
end %m index for rows in loop; *j* index for columns accounted by std2
% ———- % remove padding (before removing outliers in next section)
startr=1+b; % start row of original data (then add n-1 to get end)
xmean=xpadmean(startr:startr+n-1,:); % mean of x1D
mwSD=xpadmwSD(startr:startr+n-1,:); % movingSD for x1D
mwSD=repmat(mwSD,1,k2); % make mwSD matrix same size as x1D
x2D=x1D; %create x after 2D outliers removed (temporary as copy of x1D)
% ———- % remove 2D outliers outside lower/upper limits
t2=abs(tinv(alpha2/2,k2-1)); %2-tailed t-statistic for alpha2 and k2-1 df
mwCI=t2*mwSD; %scale SD to give moving-window CI
lower=xmean-mwCI; upper=xmean+mwCI;% lower and upper limits
x2D(x2D<=lower | x2D>=upper)=NaN; % find outliers and replace with NaN
nanCt = sum(isnan(x2D),1); % sum NaNs in each column
trialskept2(:,nanCt > 0)=[]; %reduce list of trials to trial numbers kept
x2D(:,nanCt>0)=[]; % removes columns with any NaNs (outliers) to leave x2D
%==========================================================================
